# Supplementary material for: Bioactive Secoiridoids in Italian Extra-Virgin Olive Oils: Impact of Olive Plant Cultivars, Cultivation Regions and Processing
Source: Molecules. 2021 Jan 31;26(3):743. doi: 10.3390/molecules26030743 (PMC7867028; doi:10.3390/molecules26030743)
Supplement: Supplementary file 1 [file molecules-26-00743-s001.pdf]

Supplementary Materials

Bioactive Secoiridoids in Italian Extra-Virgin Olive Oils: Impact of Olive Plant Cultivars, Cultivation Regions and Processing

Ilario Losito [1,2,*], Ramona Abbattista [1], Cristina De Ceglie [1,], Andrea Castellaneta [1], Cosima Damiana Calvano [2,3] and Tommaso R.I. Cataldi [1,2]**

[1] Dipartimento di Chimica, Università degli Studi di Bari "Aldo Moro", via Orabona 4, 70126 Bari (Italy); ilario.losito@uniba.it
[2] Centro Interdipartimentale SMART, Università degli Studi di Bari "Aldo Moro", via Orabona 4, 70126 Bari (Italy)
[3] Dipartimento di Farmacia-Scienze del farmaco, Università degli Studi di Bari "Aldo Moro", via Orabona 4, 70126 Bari (Italy)
* Correspondence: ilario.losito@uniba.it; Tel.: 0039 080 5442506
** Current address: Istituto di Ricerca sulle Acque - Consiglio Nazionale delle Ricerche (IRSA-CNR), Viale Francesco de Blasio, 5, 70132 Bari (Italy)

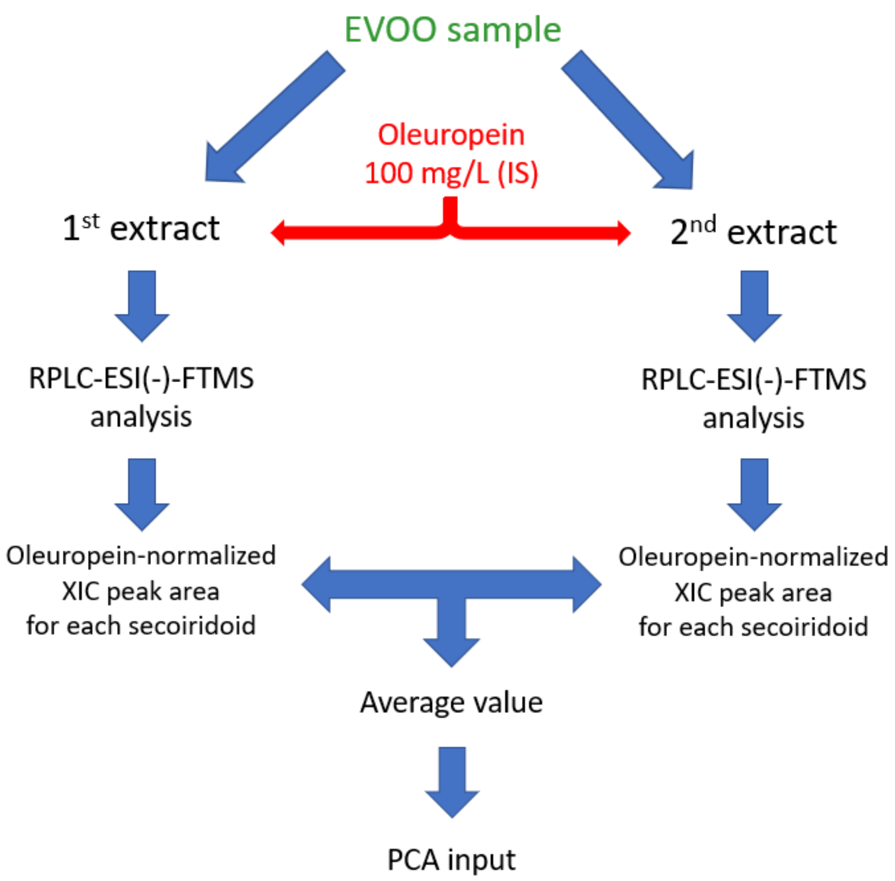

Figure S1. Outline of the procedure adopted to calculate, for each of the 60 analysed EVOO samples, oleuropein-normalized MS responses of secoiridoids that were subsequently used as variables for Principal Component Analysis (PCA). Note that, in the case of oleuropein aglycone, oleacin and oleocanthal, the oleuropein-normalized XIC peak areas of their carboxylic acid derivatives were respectively summed to those of the precursors, to account for partial oxidative degradation occurred during the storage of EVOO samples before extraction and analysis.
